# Quantitative EEG Changes in Youth With ASD Following Brief Mindfulness Meditation Exercise

**DOI:** 10.1109/TNSRE.2022.3199151

**Published:** 2022-09-02

**Authors:** Busra T. Susam, Nathan T. Riek, Kelly Beck, Safaa Eldeeb, Caitlin M. Hudac, Philip A. Gable, Caitlin Conner, Murat Akcakaya, Susan White, Carla Mazefsky

**Affiliations:** Department of Electrical and Computer Engineering, University of Pittsburgh, Pittsburgh, PA 15261; Department of Electrical and Computer Engineering, University of Pittsburgh, PA 15261 USA.; School of Health and Rehabilitation Sciences, University of Pittsburgh, Pittsburgh, PA 15260 USA.; Department of Electrical and Computer Engineering, University of Pittsburgh, PA 15261 USA.; Department of Psychology, Carolina Autism and Neurodevelopment (CAN) Research Center, University of South Carolina, Columbia, SC 29208 USA.; Department of Psychological and Brain Sciences, University of Delaware, Newark, DE 19716 USA.; Department of Psychiatry, School of Medicine, University of Pittsburgh, Pittsburgh, PA 15213 USA.; Department of Electrical and Computer Engineering, University of Pittsburgh, PA 15261 USA.; Department of Psychology, The University of Alabama, Tuscaloosa, AL 35401 USA.; Department of Psychiatry, School of Medicine, University of Pittsburgh, Pittsburgh, PA 15213 USA.

**Keywords:** Autism, EEG, mindfulness, resting-state

## Abstract

Mindfulness has growing empirical support for improving emotion regulation in individuals with Autism Spectrum Disorder (ASD). Mindfulness is cultivated through meditation practices. Assessing the role of mindfulness in improving emotion regulation is challenging given the reliance on self-report tools. Electroencephalography (EEG) has successfully quantified neural responses to emotional arousal and meditation in other populations, making it ideal to objectively measure neural responses before and after mindfulness (MF) practice among individuals with ASD. We performed an EEG-based analysis during a resting state paradigm in 35 youth with ASD. Specifically, we developed a machine learning classifier and a feature and channel selection approach that separates resting states preceding (Pre-MF) and following (Post-MF) a mindfulness meditation exercise within participants. Across individuals, frontal and temporal channels were most informative. Total power in the beta band (16–30 Hz), Total power (4–30 Hz), relative power in alpha band (8–12 Hz) were the most informative EEG features. A classifier using a non-linear combination of selected EEG features from selected channel locations separated Pre-MF and Post-MF resting states with an average accuracy, sensitivity, and specificity of 80.76%, 78.24%, and 82.14% respectively. Finally, we validated that separation between Pre-MF and Post-MF is due to the MF prime rather than linear-temporal drift. This work underscores machine learning as a critical tool for separating distinct resting states within youth with ASD and will enable better classification of underlying neural responses following brief MF meditation.

## I. Introduction

Autism spectrum disorder (ASD) is a neurodevelopmental condition characterized by challenges in social communication and restrictive and repetitive behaviors [1]. Poor emotion regulation (ER), the inability to monitor and modify emotional arousal and reactivity to engage in adaptive behavior, is as much as 7 times more common among individuals with ASD than neurotypical counterparts. Further, recent research suggests that poor ER likely underlies co-occurring mental health conditions, use of crisis services, and risk for suicide in autism [2], [3], [4], [5], [6]. Given the prevalence of ER problems in ASD, there is a need to both identify approaches to remediate ER problems and measure improvement in ER [7].

Mindfulness is an experience of present-moment and non-judgmental awareness that is cultivated through meditation practices [8]. MF meditation involves concentration exercises that are used to cultivate awareness of present moment experiences in an open and non-judgmental manner [9], [10]. Common MF meditations include awareness of breathing, walking meditation, body scans, and mindful movement. MF meditations are easily tailored to individual cognitive and functional needs with modified language and length, which is ideal for the heterogenous needs and presentations across individuals on the autism spectrum [11]. Findings across prior studies [12], [13], [14] demonstrated that interventions that utilize MF meditaitons, called mindfulness-based interventions, improved outcomes for individuals with ASD [15].

While MF appears to be a promising treatment approach in ASD, these studies have relied upon self-report mindfulness measurements that are adherent to self-interest bias and autism-specific limitations in awareness and alexithymia. Understanding neural and physological responses during MF meditations can enhance the measurement of MF as a mechaism of change in treatments. As such, this paper seeks to improve our understanding of neural responses that occur following MF meditations.

EEG has been shown to be an effective tool to investigate neurophysiological effects of MF meditation in populations without ASD [16], [17], [18]. Nyhus *et al.* [17] used EEG to demonstrate a correlation between between EEG theta oscillations and self-report MF questionnaire responses in a non-ASD sample. Qiu *et al.* [18] designed a game with conditions of varying difficulty in order to gauge frustration while using an EEG headband. They detected changes in improved game accuracy following a calming meditation exercise. Bostanov *et al.* [16] evaluated pre-post-therapy changes in event-related brain potentials (ERPs) recorded during a MF meditation task and found a correlation between the ERPs and other self-report measures of MF and meditation practice. Furthermore, a review paper from 2015 [19] collected data from 56 papers on EEG and MF meditation and found that prolonged MF practice was associated with increased alpha and theta power in healthy individuals [20] as well as patient populations.

Despite the growing empirical support of MF for improving outcomes among individuals with ASD [15], research has yet to examine the neurophysiological effects of MF meditations. Instead, autism research on MF meditations has relied on self-report measures [5]. Given the promising work using EEG methods to measure the impact of MF meditation in neurotypical populations, this study sought to apply EEG methods to measure within-session changes in ER following a single MF meditation practice among youth with ASD. Specifically, this study developed a classification framework to analyze the effect of a MF meditation exercise via brain signals of youth with ASD. The MF exercise consisted of a brief; two-minute guided awareness of breathing MF meditation completed at the middle of battery of tasks including an Affective Posner task [21]. One of the unique contributions of the presented study is investigating the neurophysiological effect of brief MF exercise, as most previous research assessed the effects a full mindfulness-based intervention, which includes several weeks of treatment and multiple different meditation exercise [20], [22].

For each participant, we collected resting-state EEG twice between tasks before the brief MF exercise and twice between tasks after the MF exercise. Our goal was to generate a classifier that would delineate between EEG resting states directly before and after the MF exercise, specifically, as a means to identify the change in neural response generated by the MF exercise. Additionally, we verified that this classifier did not relate to other instances of linear temporal order but is rather an effect of the MF exercise itself. To accomplish this, we applied the weighted sequential forward selection algorithm (WSFS) to obtain the most informative features accompanying channel locations for each participant. We then developed a classifier at the individual level that clearly separates resting-state EEG data before MF meditation exercise (Pre-MF) and after MF meditation exercise (Post-MF) within a task battery that interleaved resting state and an Affective Posner task. As a result, the classifier successfully differentiated between Pre-MF and Post-MF conditions with an average accuracy, sensitivity to Pre-MF, and sensitivity to Post-MF of 94.72%, 97.34% and 91.98%, respectively, with low variance across participants using the most informative EEG features within each participant. Additionaly, the wilxocon ranksum tests were applied on rest-states represented by individual features in order to demonstrate whether these changes generalize across participants.

## II. Methodology

### A. Participants and Experimental Setup

As part of a larger and ongoing randomized clinical trial across two sites, participants were recruited to complete EEG procedures at a baseline assessment appointment. A total of 40 participants with ASD completed EEG procedures. 5 participants were excluded due to technical issuses, since they did not have all four rest state EEG data. Inclusion criteria for the larger study were as follows: (1) ages 12–21, inclusive; (2) a clinical diagnosis of ASD, confirmed by a research reliable administration of the Autism Diagnostic Observation Schedule, Second Edition (ADOS-2)[23]; (3) presence of emotion dysregulation at an initial phone screen (Emotion Dysregulation Inventory raw score ≥ 7). All parents or guardians provided written informed consent approved by the University of Pittsburgh Institutional Review Board (IRB #STUDY17070496). During the consent and youth assent process, the participants were informed that their safety and the confidentiality of the collected data are the primary considerations. Participants were told that at any point during the experimental procedure, if they feel any discomfort, they could stop the experiment. Furthermore, all the experimental procedures described below were approved by the University of Pittsburgh Institutional Review Board which served as the single site IRB of record.

At the first site, EEG data were recorded using a Wearable Sensing DSI-24 wireless dry electrode EEG headset with 21 channels at a sampling rate of 300 Hz; channel locations are, P3, C3, F3, Fz, F4, C4, P4, Cz, A1, Fp1, Fp2, T3, T5, O1, O2, F7, F8, A2, T6, and T4 according to the international 10–20 system. The reference sensor was placed at the nominal Pz position, while ground was placed at the earlobes. At the second site, Electroencephalography (EEG) was recorded from 32 Ag/AgCl electrodes using a BrainVision actiCAP snap system (EASYCAP GmbH, Herrsching, Germany). Sensor placement was based on the international 10–20 system with a ground electrode mounted at FPz. Sensors were referenced online to the left earlobe. Data were collected using a BrainVision ActiCHamp amplifier (Brain Products GmbH, Munich, Germany) and were digitized at 500 Hz.

**Fig. 1.**
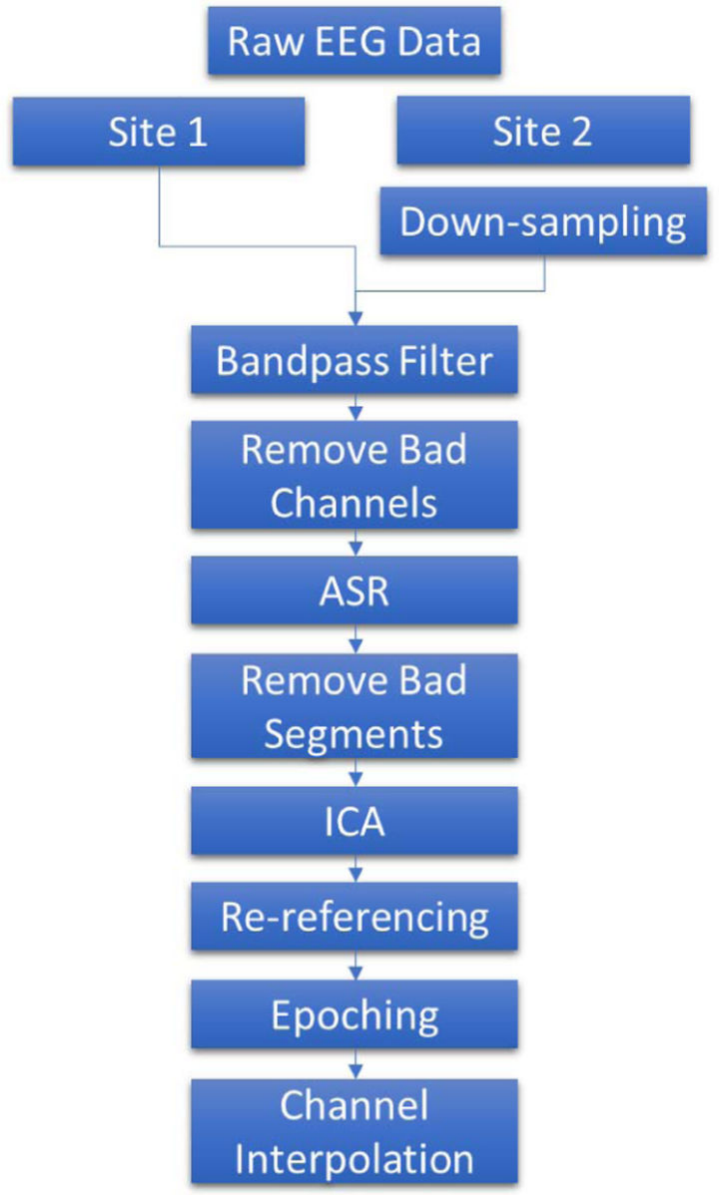
The shematic of pre-processing using EEGLAB.

All participants were seated in a comfortable chair facing a computer screen and were asked to play a card game based on an Affective Posner task. The Affective Posner task is a neuropsychological test often used to investigate the effects of covert orienting of attention in response to different cue conditions [24], [25]. The proposed Affective Posner task with deception consists of four card games with “breaks” interleaved between games in which resting state data was collected. The cards are respresented as two white squares where there is a star under one of the two white squares. During the experiment, a blue rectangle appears on one of the two white squares as a cue with the correct location of the star with 75% probability. Then, a feedback of Lose, Win, or Too Slow was presented to players based on their answer to the location of the star. Too slow feedback is provided after 60% of the correct responses in Task 3 as a deception component. Task 4 is the repitition of Task 3. Task 5 is played at the end without deception. EEG data was not collected during Task 1 or Task 2, which were used as practice rounds to learn how to play the game. In Task 1 there was Win and Lose feedback. Task 2 was the same as Task 1 with Win, Lose, and Too slow feedback. In this study, we focus on four resting state periods (see Fig. 1.A): TaskRest1 (between two Posner tasks before Pre-MF), Pre-MF (immediately before MF exercise), Post-MF (immediately following MF exercise), and TaskRest2 (between two Posner tasks after Post-MF). TaskRest1 lasted 2-minutes, while all other resting states were in 1-minute in duration. The Mindfulness condition consisted of participants listening to a MF meditation led by a MF-Based Stress Reduction teacher who has clinical expertise working with individuals with ASD (co-author). The exercise, developed specifically for this study, consisted of a 2-minute awareness of breathing task, based on Vipassana meditation practices common in MF-Based Stress Reduction. The guided practice encouraged participants to focus on physical sensations of natural breathing and non-judgmental awareness of any instances of inattention. This was delivered through an audio recording and did not include visual cues.

**Fig. 2.**
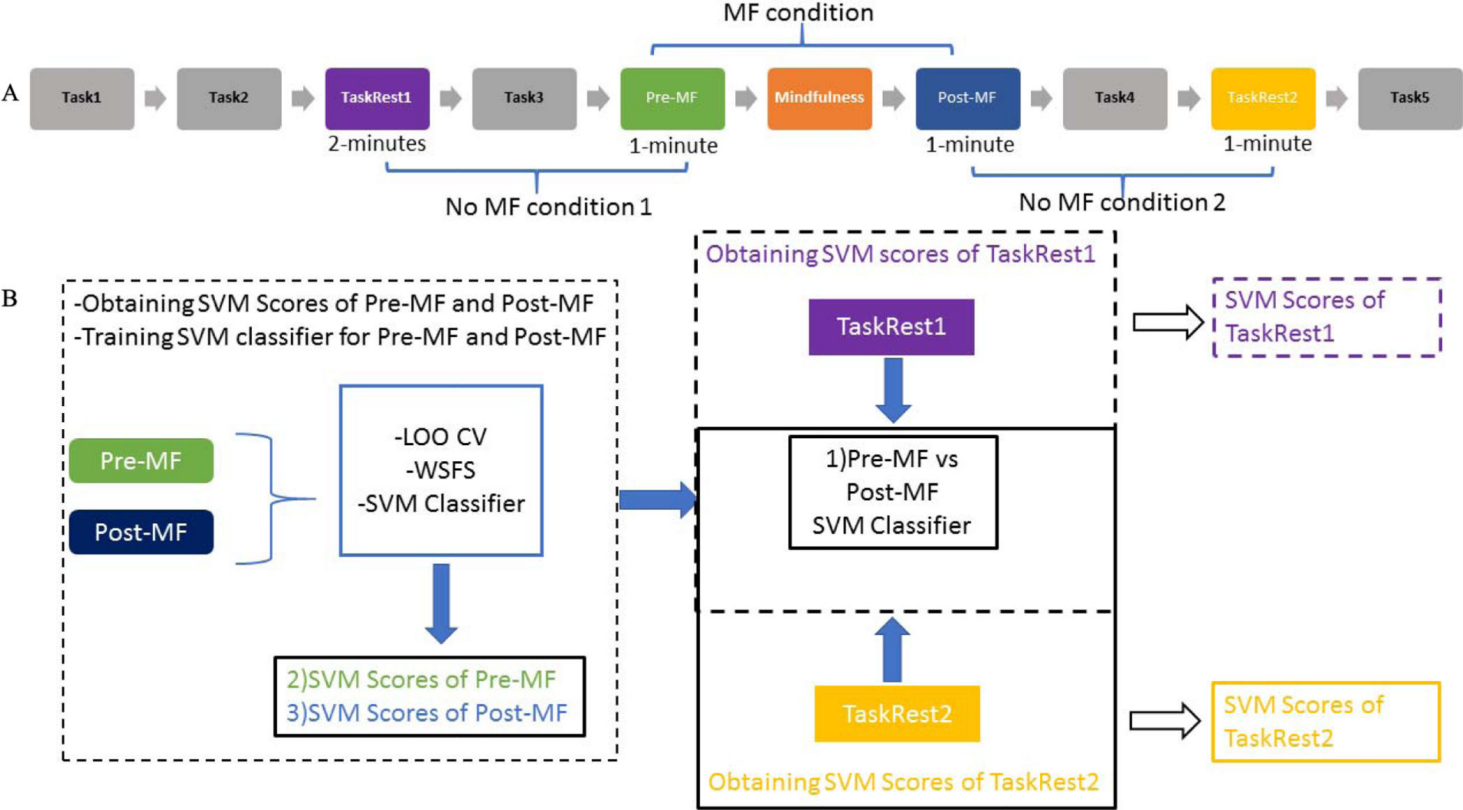
A. Order of task presentation for participants. Resting states were interweaved with the affective posner task (not addressed in the current study). B. Classification procedure framework. The SVM classifier was trained to separate Pre-MF and Post-MF resting states. The same SVM classifier was used to obtain SVM scores of TaskRest1 and TaskRest2 resting states.

In this study, we aim to investigate the changes in rest state brain activities of individuals with ASD when there is mindfulness exercise or not mindfulness exercise. As it can be seen from Fig 2.A., there was no mindfulness exercise between TaskRest1 and Pre-MF resting state, instead there is Posner Task Task3 card game. We denote this as No MF condition 1. Similarly, there is no mindfulness exercise between Post-MF and TaskRest2 resting state but there is Posner Task Task4 card game between Post-MF and TaskRest2 resting state which we denote as no MF condition 2. In contrary, there is a mindfulness exercise between Pre-MF and Post-MF rest state, and we denote this as MF condition

### B. Pre-Processing With EEGLAB

Pre-processing of EEG data was performed using EEGLAB(v2022.0)[26] within MATLAB R2021b. The pre-processing pipeline is shown in Fig.1.

First, the data collected from the second site is down-sampled to 300 Hz. Then, a finite impulse response (FIR) Kaiser-windowed band-pass filter with cut-off frequencies of 1 and 30 Hz was used to filter the resting-state EEG data collected from both sites. Bad EEG channels were removed if they had no signal for at least 5 seconds or if the line noise relative to the channel signal was greater than 4 standard deviations from the signal mean. After channel removal, artifact subspace reconstruction (ASR) was completed. ASR corrects bad EEG signal segments by comparing clean portions of the data to the rest of the data [27]. PCA is used and for any components’ standard deviations greater than 30 times the standard deviations from the clean components, those components are rejected. Next, any data segments that exceed the mean power by more than 20 standard deviations within atleast one quarter of the EEG channels are removed. All EEGLAB parameters were selected using visual inspection of the data.

Then, Infomax independent component analyses (ICA) was applied to remove additionalartifacts [28], [29]. Any independent components that were identified as brain activity with at least 70\% confidence, using the ICLabel classifier in EEGLAB [30], were kept and all other components were removed After that, the EEG data was re-referenced to the mean of channels A1 and A2. Next, the resting state EEG data was segmented into one-second epochs. Lastly, channel data that was removed during pre-processing was interpolated using spherical spline interpolation.

### C. Feature Extraction

Feature extraction was performed for all four resting states (see Fig.2.A). Spectral features were calculated using Welch’s periodogram method [31] for each channel; P3, C3, F3, Fz, F4, C4, P4, Cz, Fp1, Fp2, T3, T5, O1, O2, F7, F8, T6, T, Pz. A total of seven spectral features were calculated for each epoch and each channel. The spectral EEG features calculated for each channel are as follows: (1) the total power across the spectral range of this study (4–30 Hz); the total power in the (2) Theta (4–7 Hz), (3) Alpha (8–12 Hz), and (4) Beta (16–30 Hz) frequency bands; the relative power (i.e., unique contribution of each band) computed as the ratio of spectral band to overall total power in the (5) Theta, (6) Alpha, (7) and Beta frequency bands [32].

### D. Separation of Resting States Based on Individual Feature Comparison

The Wilcoxon rank-sum test with a significance level of 0.05 was conducted to investigate whether there is a significant difference between and across resting states represented by each spectral feature in overall channels, and at each brain region. The Wilcoxon rank test is a nonparametric hypothesis test where the alternative hypothesis states that both classes come from different distributions [33]. More specifically, each spectral feature is averaged over all channels and averaged over each brain region for each trial across participants to form a vector for each rest state separately. Then, we performed a Wilcoxon rank-sum test over these feature vectors to investigate the significance between (i) TaskRest1 vs Pre-MF, (ii) TaskRest1 vs Post-MF, (iii) TaskRest1 vs TaskRest2, (iv) Pre-MF vs Post-MF, (v)Pre-MF-TaskRest2 and (vi) Post-MF vs TaskRest2 rest state represented by each feature over all channels, and at each brain region: frontal (F3, F4, F7, F8), midline (Cz, Pz, Fz), prefrontal (Fp1, Fp2), parietal (P3, P4), occipital (O1, O2), temporal (T3, T4, T5, T6), and central (C3, C4).

### E. Machine Learning: Feature Selection and Classification

The features described in section 2.2 were normalized to have a mean of zero using z-score normalization across all epochs at the individual level and concatenated to form a feature vector. After normalization, the weighted sequential feature selection (WSFS) algorithm was used to obtain the most informative features at identified channel locations for each participant. The WSFS algorithm is a feature selection technique which uses a bottom-up search starting from an empty set of features and gradually adds features that maximize classifier performance [21]. More specifically, the cost function of the WSFS algorithm was set up to maximize the classification rate of the radial basis kernel support vector machine (RBF SVM) classifier over all feasible feature subsets while maintaining a balance between correct classification rates of Pre-MF and Post-MF. In this step, 10-fold cross validation was adopted to train the RBF SVM classifier with a chance level of 50% with 5 Monte Carlo simulations in order to find the most robust subset of features without overfitting for each participant. Once the most informative features for each subject were selected, a RBF SVM classifier was first trained to identify significant differences between the resting state EEG data before (Pre-MF) and after (Post-MF) a MF meditation exercise as seen in the first step of Fig. 1.B. 5-fold cross validation was applied to overcome overfitting. Then, we conducted 100 Monte Carlo runs (each with different random initialization) for each participant. Next SVM scores of Pre-MF and Post-MF resting states were calculated by averaging over 100 Monte Carlo runs.

In the second step of Fig. 1.B, the pre-trained classifier was used in the classification of TaskRest1 versus Pre-MF and for the classification of Post-MF versus TaskRest2 to obtain SVM scores of TaskRest1 and TaskRest2.

Additionally, we applied a two-sided Wilcoxon rank-sum test with a significance level of 0.05 across the two sites using the classification accuracies, sensitivities, specificity in order to test whether there is a statistical significance across data collection sites. The two-sided Wilcoxon rank test is a nonparametric hypothesis test where the alternative hypothesis states that both classes come from different distributions [33].

### F. Validation

Next, we validated that the separation between the resting state EEG data before and after MF-meditation exercise was a result of the MF exercise itself (Fig. 1B). Thus, we sought to show that the separation between Pre-MF and Post-MF (i.e., distinctiveness of MF classification) is greater than the separation between any other two consecutive resting state EEG periods (e.g., TaskRest1 versus Pre-MF; Post-MF versus TaskRest2). We used the SVM scores generated by the classifier to calculate the separation between each set of two consecutive resting states. We first calculated the probability distribution of SVM scores of all resting states through kernel density estimation at the individual level. Next, we investigated if there is any separation between resting states using the distribution of SVM scores of resting states. Specifically, we calculated the distance between resting states using the learned kernel density-based estimations and symmetric Kullback Leibler (Sym KL) distance metric, which is applicable to compare two probability density functions [34]. In following, using the probability density functions of SVM scores of resting state *P_SVM_ BSL_i_*, and resting state *i* (i = 1,2,3,4: TaskRest1, Pre-MF, Post-MF, and TaskRest2), we formulated the symmetric Kullback Leibler distance measurement between baselines (1), as shown at the bottom of the page.

Using the KL distance metric, we calculated the distances between Pre-MF and Post-MF (*D_SVM_ (BSL*2| *BSL*3)), between TaskRest1 and Pre-MF (*D_SVM_ (BSL*1| *BSL*2)), and between Post-MF and TaskRest2(*D_SV M_ (BSL*3| *BSL*4)). These are shown in equations (2) and (3), as shown at the bottom of the page.

Finally, we calculated the probability density functions of *Distance*1 and *Distance*2 using a normalized histogram across all individuals to investigate the probability that the separation between Pre-MF and Post-MF is larger than the separation between any other two resting states. Note that Distance1 is used to compare the separation between Pre-MF and Post-MF with the separation between TaskRest1 and Pre-MF. Furthermore, Distance2 is used to compare the separation between Pre-MF and Post-MF with the separation between Post-MF and TaskRest2. Distance1 and Distance2 take values between −1 and 1. When Distance1 is positive it indicates that the effect of MF (i.e., the distance between Pre-MF and Post-MF) is greater than the effect of linear time at the beginning of the experiment (i.e., distance between TaskRest1 and Pre-MF). Likewise, Distance2 is positive when the distance between Pre-MF and Post-MF is greater than the distance between Post-MF and TaskRest2.

## III. Results

The results of the Wilcoxon rank-sum test to investigate the significant differences between between rest states, represented by each spectral feature averaged over all channels, is presented in Table I.

The results of the Wilcoxon rank-sum test applied to each spectral feature at each brain region is reported for Pre-MF vs Post-MF in Table II, TaskRest1 vs Post-MF, TaskRest1 vs TaskRest2, Pre-MF vs Post-MF, Pre-MF-TaskRest2, and Post-MF vs TaskRest2 in Table S1 in the supplementary document.

**Fig. 3.**
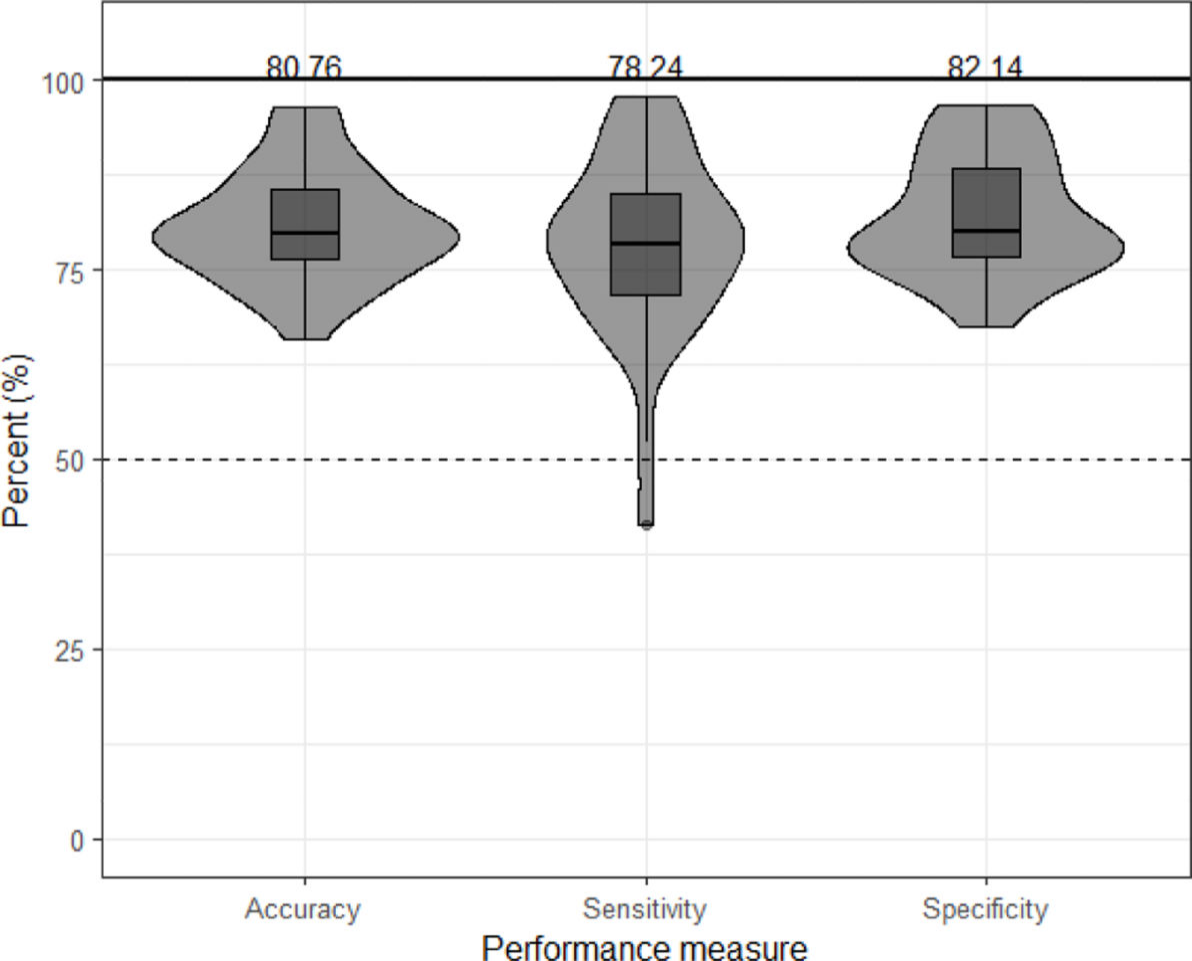
The average performance measurements of Pre-MF vs Post-MF classifier.

**Fig. 4.**
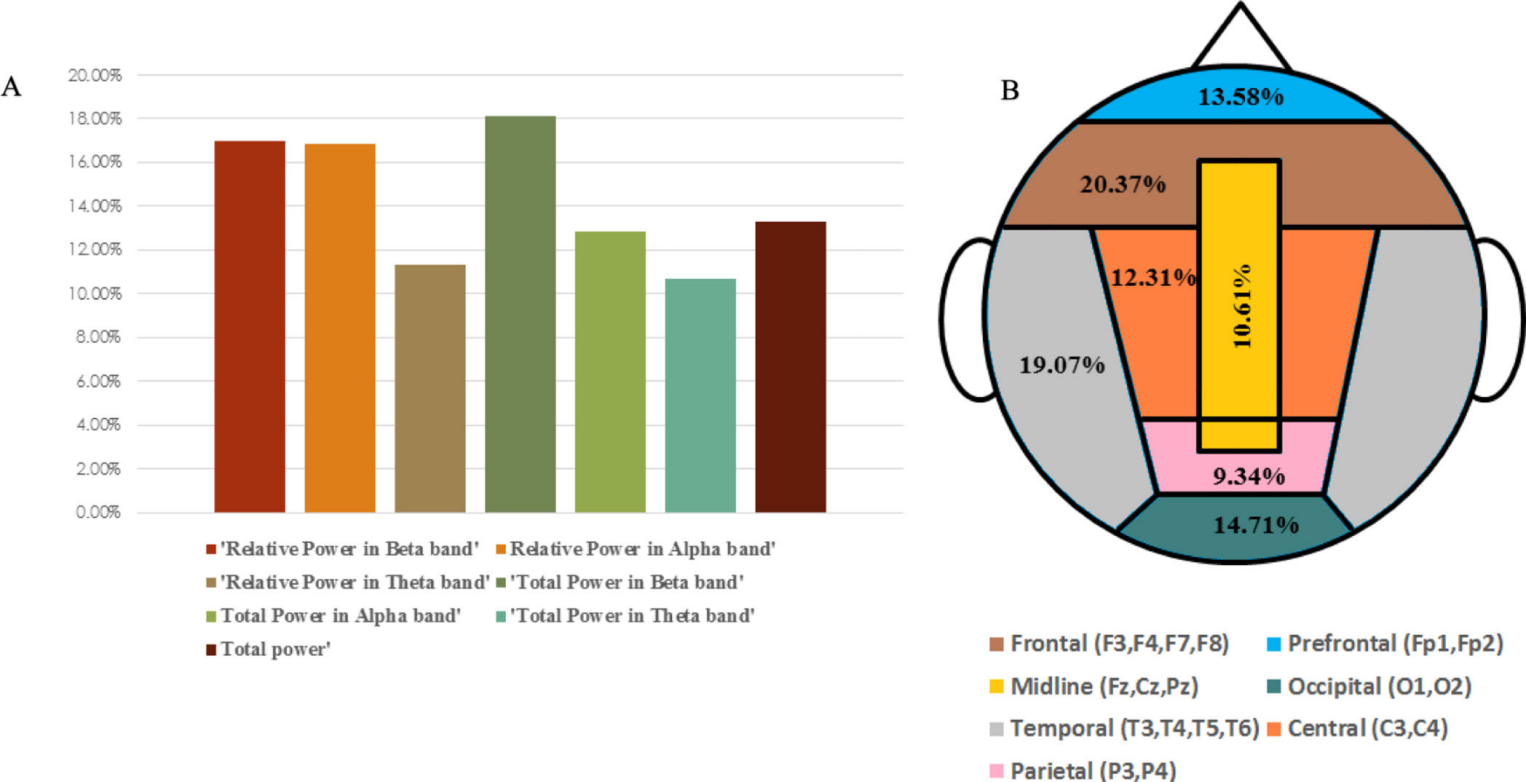
A) The percentage of relative occurrence of the spectral features across participants in the classification of Pre-MF and Post-MF. B) The percentage of relative occurrence of the channels across participants in the classification of Pre-MF and Post-MF.

**Fig. 5.**
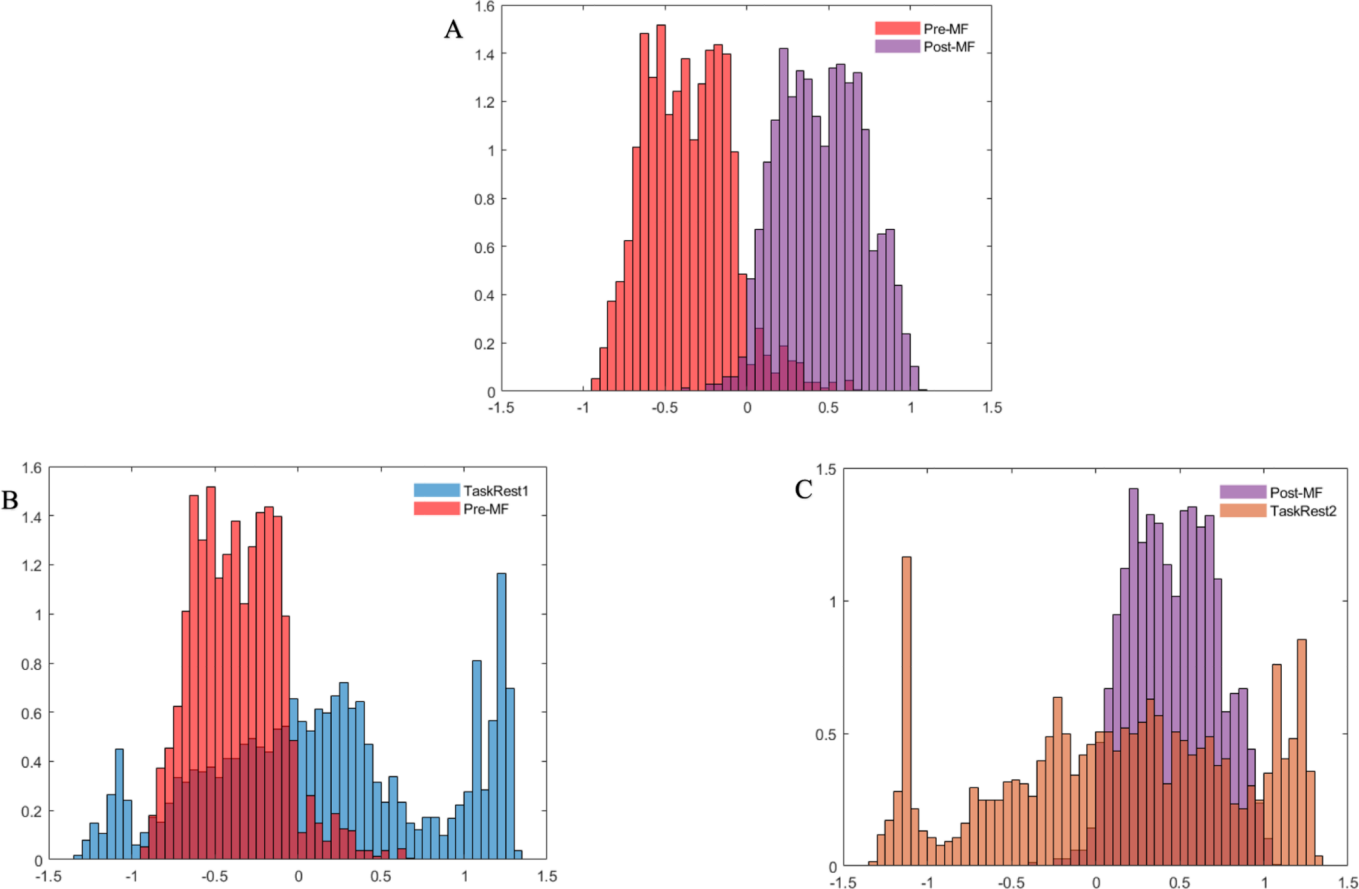
The distribution of SVM scores of resting-states across participants A. illustrates the distribution of SVM scores of Pre-MF and Post-MF. B. demonstrates separability between distribution of SVM scores of TaskRest1 and Pre-MF while C. depicts the separation between the distribution of SVM scores of Post-MF and TaskRest2.

Then, we present the performance of the RBF kernel SVM classifier in terms of accuracy, Pre-MF sensitivity, and Post-MF sensitivity. Next, we provide a bar graph (Fig. 3)

(1) $$D_{SVM}(BSLi|BSLj),\;i,\;j=1,2,3,4,\;i\neq j\;D_{SVM}(BSLi|BSLj)\;\;=\frac{1}{2}\sum_{x\in X}\left[P_{SVM}BSL_{i}(x)\log\left(\frac{P_{SVM}BSL_{i}(x)}{P_{SVM}BSL_{j}(x)}\right)+P_{SVM}BSL_{j}(x)\log\left(\frac{P_{SVM}BSL_{j}(x)}{P_{SVM}BSL_{i}(x)}\right)\right]$$

(2) $$Distance\;1=\left[\frac{D_{SVM}(BSL2|BSL3)}{D_{SVM}(BSL1|BSL2)+D_{SVM}(BSL2|BSL3)}-\frac{D_{SVM}(BSL1|BSL2)}{D_{SVM}(BSL1|BSL2)+D_{SVM}(BSL2|BSL3)}\right]$$

(3) $$Distance\;2=\left[\frac{D_{SVM}(BSL2|BSL3)}{D_{SVM}(BSL2|BSL3)+D_{SVM}(BSL3|BSL4)}-\frac{D_{SVM}(BSL3|BSL4)}{D_{SVM}(BSL2|BSL3)+D_{SVM}(\text{BSL3|BSL4)}}\right]$$

and topographic map (Fig. 4) to show the features and brain regions that contributed most frequently to the classification between Pre-MF and Post-MF. Finally, we validate our proposed classification scheme to indicate the separation of Pre-MF and Post-MF is a result of MF by comparing the distributions of SVM scores for the effect of MF (Pre-MF vs Post-MF), early linear time shift (TaskRest1 vs Pre-MF), and late linear time shift (Post-MF vs TaskRest2 ) through the use of histograms (Fig. 5) of Distances 1 and 2 as developed in equations (2) and (3).

Table I indicates the results of the Wilcoxon rank-sum test applied on each spectral feature averaged over all channels between resting states. The Wilcoxon rank-sum test over Pre-MF and Post-MF rest state represented by each spectral features averaged over all channels indicated that total alpha, total power, total theta, and relative alpha band were significantly higher in the Post-MF resting state with p-values of 1.0848e-12, 0.00349, 0.00033 and 1.92e-18, respectively. Total beta band power and relative beta band power were significantly higher in Pre-MF than Post-MF with p-values of 6.22e-23 and 9.34e-31, while relative theta band power did not show any significant differences in comparison between Pre-MF and Post-MF. Moreover, across the above-mentioned six statistical tests, we observed that total alpha, total power, and relative alpha band power indicated a significant increase from TaskRest1 to Pre-MF. Then, the increase reached a maximum in the Post-MF resting state, followed by a significant decrease in the end (TaskRest2). Total beta and relative beta band indicated a significant increase from TaskRest1 to Pre-MF. Then, the total beta and relative beta band power decreased to a minimum in Post-MF followed by a significant increase in TaskRest2.

The result of the Wilcoxon rank-sum test over Pre-MF vs Post-MF rest state represented by each spectral feature averaged within brain regions is represented in Table II. In this table, we observed that total alpha and relative alpha band power was significantly higher in Post-MF than Pre-MF in all brain regions. Similarly, total beta and relative beta band power was significantly higher in Pre-MF than Post-MF in all brain regions. Moreover, relative theta power band did not show any significant differences at temporal, prefrontal, or central regions while relative theta power band was significantly higher in Post-MF at occipital and frontal regions and higher in Pre-MF at midline and parietal regions. Total power at frontal and occipital regions did not show any significant differences in Pre-MF vs Post-MF, but was significantly higher at midline, temporal, parietal, prefrontal and central regions in Post-MF than in Pre-MF.

Additionally, we investigated the behavior of each averaged spectral feature at brain regions across resting states by performing the Wilcoxon rank-sum test between TaskRest1 vs Pre-MF, TaskRest1 vs Post-MF, Pre-MF vs Post-MF, TaskRest1 vs TaskRest2, Pre-MF-TaskRest2, and Post-MF vs TaskRest2 resting states, as reported in Table S1 in the supplementary document. As a result, total alpha band power at frontal, central, midline, parietal, temporal, and occipital regions indicated a significant increase from TaskRest1 to Post-MF, followed by a significant decrease in TaskRest2. However, total alpha power at the prefrontal region indicated a trend that remains the same in significance from TaskRest1 to Pre-MF, followed by a significant increase in Post-MF and a significant decrease in the end (TaskRest2).

The propagation of total power across rest states of TaskRest1, Pre-MF, Post-MF and TaskRest2 follows a trend that there was a significant increase from TaskRest1 to Pre-MF, with a significant increase, reaching a maximum in Post-MF, followed by a significant decrease in TaskRest2 at midline, temporal, parietal, central while there was no change in significance level in TaskRest2 at prefrontal brain region. Total power at prefrontal brain regions indicated a significant increase from TaskRest1 to Post-MF, then remain in the same significant level in TaskRest2.

Total power at the occipital region indicated a significant increase from TaskRest1 to Pre-MF. Then, the trend did not change in the significance level through Pre-MF to Post-MF but decreased in significance level in the end.

On the contrary, total theta band power at midline, parietal, occipital, and central brain regions did not indicate any pattern across resting states but total theta power band at frontal, prefrontal and temporal regions indicated a trend that there was no difference in significance level from TaskRest1 to Pre-MF followed by a significant increase in Post-MF. Then, the trend pursued a significant decrease in TaskRest2.

Relative theta band power at temporal and central regions indicated a significant decrease from TaskRest1 to Pre-MF and remained at the same significance level until the end (TaskRest2). Relative theta band power showed a trend that there was a significant decrease from TaskRest1 to Pre-MF with a significant increase in Post-MF, followed by a significant increase at the occipital region in TaskRest2 while the trend remained the same significance in TaskRest2 at the frontal brain region. Additionally, Relative theta at midline and parietal brain regions indicated a significant decrease from TaskRest1 to Post-MF followed by a significant increase in TaskRest2. However, we did not observe any pattern of relative theta band power in prefrontal across rest states.

Relative alpha band power at midline, temporal, parietal, frontal, and central brain regions indicated a significant increase from TaskRest1 to Post-MF with a significant decrease in TaskRest2 while the behavior of relative alpha band power did not change in significance level from TaskRest1 to Pre-MF followed by an increase in Post-MF and decrease in TaskRest2 at prefrontal and occipital brain regions.

Total beta power at central, midline, temporal, frontal, and occipital brain regions indicated a significant increase from TaskRest1 to Pre-MF with a significant decrease in Post-MF, followed by a significant increase at the end. Total beta band power at parietal and prefrontal brain regions remained at the same significance level from TaskRest1 to Pre-MF. Then, there was a significant decrease in Post-MF followed by a significant increase in TaskRest2.

Relative beta band power at temporal, central, frontal, and occipital brain regions show that there was a significant increase from TaskRest1 to Pre-MF with a significant decrease in Post-MF, followed by a significant increase in TaskRest2. On the other hand, relative beta band power at parietal brain region indicated a significant decrease from TaskRest1 to Post-MF followed by a significant increase in the TaskRest2. Relative beta band power at prefrontal brain region remained the same level significance from TaskRest1 to Pre-MF, Then, the trend decreased in significance level in Post-MF, followed by increase in significance level in TaskRest2. Relative beta band power in midline remained in the same significance level from TaskRest1 to Pre-MF. Then the trend showed a significant decrease in Post-MF followed by a significant increase in TaskRest2

As we described above, the proposed classification scheme summarized in Fig. 2.B was used to identify significant difference between the resting state EEG data before and after a MF meditation exercise. Recall that, the overall classification and validation framework includes three main parts (i) classification of EEG data before (Pre-MF) and after (Post-MF) the MF meditation exercise using an RBF SVM classifier; (ii) Obtaining SVM scores of TaskRest1 and TaskRest2 using the trained SVM classifier generated from step i (iii) Calculating the symmetric Kullback Leibler (Sym KL) distance metric of the distance between SVM scores of resting states to validate the MF prime.

The performance metrics include the accuracy, sensitivity, specificity and F1 scores. As seen in Fig. 3, the average accuracy is 80.76 % (range 65.89–96.36%) while the average sensitivity (correct identification of Pre-MF) is 78.24% (range 41.33–97.74%). The average specificity (correct identification of Post-MF) is 82.14% (range 67.60%−96.84%) for all participants. F1-scores indicate an average value of 0.79 (range 0.50–0.96) across all participants. The performance measurements of Pre-MF and Post-MF classification by the RBF SVM classifier are presented for each participant in Table S2 in the supplementary document. Furthermore, we applied the two-sided Wilcoxon rank-sum test on the classification accuracies, sensitivities, and specificity between the two testing sites. The results indicated that there was no significant difference across sites using classification accuracy, sensitivity, specificity with p-values of 0.485, 0.073, and 0.5354, respectively. This means that we fail to reject the null hypothesis (that the data between the two sites come from different distributions) and therefore we combined the data across sites for our analysis. From applying the WSFS algorithm during the classification, we found the most significant features in performing Pre-MF versus Post-MF classification within each participant. Notably, all features contributed to the classifier, with a minimum occurrence of 10.70% (Total Power in Theta band).

The percentage of the relative occurrence of the spectral features and channels across participants is illustrated in Fig. 4. Fig. 4.A indicates that total power contributed to 13.27% of all significant features. Total power in the Beta band also had a high significance of 18.08%. The total power in the Theta and Alpha frequency bands contributed to the classification with 10.70% and 12.85%, respectively. The relative power in the Beta band contributed 16.98% and the relative power in the Theta band contributed 11.29% while the relative power in the Alpha band contributed 16.84% to the classification. Fig. 4.B shows the percent contribution of the most prominent channel regions across participants in the classification of Pre-MF versus Post-MF. The frontal channels (F3, F4, F7, F8) and temporal channels (T3, T4, T5, T6) showed the highest percentage of contribution in the classification of Pre-MF versus Post-MF with 20.37% and 19.07%, respectively across participants. The distribution of the selected features for each individual for Pre-MF and Post-MF classification problem is shown in the supplementary document in Table S3.

To provide further insight into resting state EEG separation before and after MF exercise classification (Pre-MF vs Post-MF classification). The distribution of SVM scores is plotted in Fig. 5. In this figure, the X-axis indicates SVM score values while the y-axis indicates the frequency of the SVM score values. Fig. 5.A depicts the separation between the distribution of SVM scores for Pre-MF and Post-MF. The separation between the distribution of SVM scores of TaskRest1 and Pre-MF can be found in Fig. 5.B and Fig. 5.C demonstrates the distinction between the distribution of SVM scores of Post-MF and TaskRest2. As anticipated, Fig. 5 indicates that there is a larger separation between Pre-MF and Post-MF (MF condition) and both smaller separation between TaskRest1 and Pre-MF (No MF condition 1) and smaller separation between Post-MF and TaskRest2 (No MF condition 2). To get a numerical value of resting state EEG separation, we calculated the Sym KL distance metric as described in equations (2) and (3) in Section 2.5. We calculated that probabilities of Distances 1 and 2 being greater than zero in order to show that the separation between Pre-MF and Post-MF is larger than the separation between any two other baselines. Specifically, our results indicated that the separation between Pre-MF and Post-MF (MF condition) is higher than both the separation between TaskRest1 and Pre-MF (No MF condition 1) and the separation between Post-MF and TaskRest2 (No MF condition 2) with probabilities of 0.6286 and 0.8857, respectively. Thus, these probabilities are above chance (0.5) and indicate that the classifier describes an effect of the MF meditation exercise rather than temporal-linear drift (i.e., procedural order effect).

## iV. Discussion

The purpose of our research was to establish a classification system that could effectively measure if a brief MF meditation exercise influenced brain activity by finding separation between resting state EEG data before and after the MF meditation practice. We have obtained comprehensive results showing that our proposed classification framework, using a non-linear combination of selected EEG-features from selected channel locations, distinguished Pre-MF and Post-MF resting states with an average accuracy, sensitivity, and specificity of 80.76%, 78.24% and 82.14%, respectively across all participants. Additionally, we found frontal channels and temporal channels, and total power in beta band (16–30Hz), and relative power in alpha band (8–12Hz) and total power (4–30 Hz) to be most informative in separating before and after MF meditation exercise.

Next, we investigated each averaged raw-EEG feature over all channels by applying Wilcoxon rank-sum tests between Pre-MF and Post-MF resting states. As a result, total alpha, total theta, relative alpha band, and total power were significantly higher in Post-MF while total beta band and relative beta band were significantly higher in Pre-MF. However, relative theta band power did not show any significant difference in Pre-MF vs Post-MF rest state. After, we investigated each averaged raw-EEG feature over brain regions and found that total alpha band and relative alpha band, and total power were significantly higher in Post-MF while total beta band and relative band power were significantly higher in Pre-MF rest state at all brain regions. On the other hand, relative theta band power did not show any significant difference at temporal and prefrontal and central brain regions, and total power did not indicate a significant difference at frontal and occipital brain regions in Pre-MF vs Post-MF but was significantly higher at midline, temporal, parietal, prefrontal and central regions in Post-MF than in Pre-MF.

Furthermore, we investigated the feature propagation averaged over all channels across resting states by performing Wilcoxon rank-sum tests between (i)TaskRest1 vs Pre-MF, (ii) TaskRest1 vs Post-MF, (iii) TaskRest1 vs TaskRest2, (iv) Pre-MF vs Post-MF, (v) Pre-MF-TaskRest2 and (vi) Post-MF vs TaskRest2. The propagation of total alpha, relative alpha, and total power band across resting states share a common trend of a significant increase from TaskRest1 to Post-MF, followed by a significant decrease in TaskRest2. In contrast, the total beta and relative beta band power indicated a significant increase from TaskRest1 to Pre-MF with a significant decrease in Post-MF, followed by a significant decrease in TaskRest2.

The propagation of each feature averaged over frontal brain region across rest states indicated a trend that relative beta and total beta band power with significant increase from TaskRest1 to Pre-MF. The trend decreased in Post-MF, followed by a significant increase in TaskRest2. Relative alpha and total alpha power averaged over frontal brain region indicated that there was a significant increase from TaskRest1 to Pre-MF with a significant increase in Post-MF, followed by a significant decrease in TaskRest2. Relative theta power averaged over frontal brain region indicated that there was a significant decrease from TaskRest1 to Pre-MF with a significant increase in Post-MF while the trend remained the same significance in TaskRest2 at the frontal brain region. Total power averaged over frontal brain region showed a significant increase from TaskRest1 to Pre-MF while the trend remained the same significance from Post-MF to TaskRest2. Total theta band power indicated a trend that remained the same significance from TaskRest1 to Pre-MF while the trend decreased from Post-MF to TaskRest2. The propagation of total power, total alpha band power and relative alpha band power averaged over temporal brain region indicated that there was a significant increase from TaskRest1 to Pre-MF with a significant increase in Post-MF, followed by a significant decrease in TaskRest2. Conversely, the total beta relative beta power showed a significant increase from TaskRest1 to Pre-MF with a significant decrease in Post-MF, followed by a significant decrease in TaskRest2 at the temporal brain region. Total theta band power indicated a trend that remained the same significance from TaskRest1 to Pre-MF. Then, the trend increased in Post-MF followed by a significant decrease in TaskRest2. Relative theta power showed that there was a significant decrease from TaskRest1 to Pre-MF while the trend remained the same significance from Post-MF to TaskRest2 at the temporal brain region. Overall, we did not find any significance in relative theta band power averaged over brain regions of temporal, prefrontal and central brain regions by performing Wilcoxon rank-sum tests in Pre-MF vs Post-MF rest states. Similarly, the relative theta band power averaged over all channels did not show any significance by applying Wilcoxon rank-sum test over Pre-MF vs Post-MF rest states. On the other hand, our classification technique indicated a clearer separation between resting states before and after the MF meditation exercise since SVM scores are a result of nonlinear transformations of the above-mentioned features.

Finally, we validated our proposed classification scheme describes an effect of the MF meditation exercise rather than temporal-linear drift using the symmetric Kullback Leibler (Sym KL) distance metric to measure the distance between SVM scores for resting states. To find separation between the resting states, we first performed feature selection, which optimized the performance of the RBF SVM classifiers. The benefit of performing feature selection at the individual level is that it enhances classification results without requiring features to be significant among all participants. Although group trends can be insightful, each participant is different and tailoring classification to each individual will provide the best results. Furthermore, RBF SVM classifiers were used because all features themselves were not separable in terms of different resting state EEG data. The classifiers generate non-linear combinations of features which are separable, also making the SVM score output of the classifiers separable. Another benefit of using classification is that it inherently denoises the EEG data. Any random effects seen in an individual EEG trial are effectively ignored since the classifier is trained to find trends across all EEG trials.

This project sought to develop an objective measurement of MF meditation in ASD, given the limitations of using self-report measurement of MF in ASD. This approach has the potential to strengthen the scientific measurement and findings of using MF meditations with individuals and families with ASD. We observed the most significant changes in frontal and temporal region activity before and after the MF meditation. In addition, total beta band power and relative theta band power and total power contributed significantly to the classification of before and after MF meditation exercise. This aligns with previous research and shows consistency between the effects of MF in previous research on typically developing individuals and other psychiatric conditions [19], [22]. Previous research showed that MF resulted in increases in the alpha power band and theta band for typically developing populations. Similarly, in our study, we found that alpha band power and theta band power resulted in a significant increase due to 2-minutes brief mindfulness meditation exercise for individuals with autism spectrum disorder by performing Wilcoxon rank-sum test over Pre-MF vs Post-MF rest state represented by these features averaged over all channels (See Table I.)

While the aforementioned studies provide evidence for EEG data as a sensitive measure to assess the impact of MF meditation in neurotypical populations, there is a notable variability in both the duration of the MF exercise and the inclusion of healthy control groups. In literature, most of the brief mindfulness intervention studies ranged from less than 5 to 25 min in length, with an average length of 15 min [35]. To our knowledge, we are the first to investigate the impact of a brief two-minute MF meditation exercise in an ASD population by detecting the changes in EEG.

Our study has three main limitations. The first one is that we did not have a control group. This affects the ease of showing changes in EEG were in fact due to the MF meditation exercise. However, we illustrated the validation of MF exercise prime rather than linear drift using the symmetric Kullback Leibler (Sym KL) distance metric of the distance between SVM scores for resting states as described in Section 2.5. The second is that the study design limits the ability to make conclusions on the lasting impact of the brief MF meditation, as participants only completed the study procedures and brief mindfulness exercise once. Finally, this work was completed on a small sample of youth with ASD, and we are not able to draw conclusions for other age groups. However, we do anticipate that the classification system developed can be utilized in future work with larger samples and age groups. Future work might explore the effects of different durations of MF meditation exercises as well as the impact of regular meditation practice versus occasional meditations

## V. Conclusion

In summary, we developed a classification system that is able to detect changes in resting state EEG behavior before and after a brief MF meditation exercise designed specifically for youth with ASD. We found that total power and total power in beta band, total power in theta band showed a high significance in classification of before and after MF meditation exercise. The results of this study suggest that MF meditations, even brief, may have promising impact for youth with ASD and warrants further exploration. While our findings are promising, it still remains an open question if these detected neural responses will translate to more global outcomes in improved mental health symptoms and adaptive behaviors. We demonstrated that machine learning can be used to better find the separability between resting states. In future work, we aim to identify sub-groups of individuals with ASD that share similar features at certain electrode locations that help distinguish resting state EEG before and after MF exercise. Furthermore, we aim to develop investigate the trial-by-trial behavior/propagation of the selected features and non-linear combination of these features during resting states and MF exercise as a potential means to investigate changes in EEG due to changes in impaired emotional regulations.

## Supplementary Material

supp1-3199151

## Figures and Tables

**TABLE I:** The Results of Wilcoxon Rank-Sum Test Applied on Each Spectral Feature Averaged Over All Channels Between Rest States

Features	TaskRest1 vs Pre-MF	TaskRest1 vs Post-MF	TaskRest1 vs TaskRest2	Pre-MF vs Post-MF	Pre-MF vs TaskRest2	Post-MF VS TaskRest2
**Total Alpha Band Power**	Pre-MF > TaskRest1 (p=1.66E-15)	Post-MF > TaskRest1 (p=7.82E-514)	TaskRest4 > TaskRest1 (p=1.06E-ll)	Post-MF > Pre-MF (p=1.08E-12)	p=0.24946	Post-MF > TaskRest2 (p=2.19E-16)
**Total Beta Band Power**	Pre-MF > TaskRest1 (p=4.97E-11)	Post-MF < TaskRest1 (p=2.00E-05)	TaskRest4 > TaskRest1 (p=2.85E-07)	Post-MF < Pre-MF (p=6.22E-23)	p=0.33804	Post-MF < TaskRest2 (p=2.37E-17)
**Total Power**	Pre-MF > TaskRestl (p=2.21E-28)	Post-MF > TaskRest1 (p=1.80E-44)	TaskRest4 > TaskRest1 [p=7.80E-23)	Post-MF > Pre-MF (p=0.00349)	p=0.37948	Post-MF > TaskRest2 (p=0.00020)
**Total Theta Band Power**	(p=0.46511)	Post-MF > TaskRest1 (p=0.00153)	p=0.46997	Post-MF > Pre-MF (p=0.00033)	p=0.98968	Post-MF > TaskRest2 (p=0.00069)
**Relative Theta Band Power**	Pre-MF< TaskRest1 (p=1.25E-14)	Post-MF < TaskRest1 (p=6.51E-14)	TaskRest4 < TaskRest1 (p=5.30E-09)	p=0.71977	p=0.12599	p=0.23293
**Relative Alpha Band Power**	Pre-MF > TaskRestl (p=6.90E-07)	Post-MF > TaskRestl (p=1.10E-42)	TaskRest4 > TaskRestl (p=0.00020)	Post-MF > Pre-MF (p=1.92E-18)	p=0,20870	Post-MF> TaskRest2 (p=1.62E-23)
**Relative Beta Band Power**	Pre-MF > TaskRest1 (p=9.67E-05)	Post-MF < TaskRest1 (p=5.73E-20)	TaskRest4 > TaskRest1 (p=0.00067)	Post-MF < Pre-MF (p=9.34E-31)	p=0.79575	Post-MF< TaskRest2 (p=3.79E-27)

**TABLE II:** The Results of Wilcoxon Rank-Sum Test Applied on Each Spectral Feature Averaged Over Each Brain Regions of Frontal, Midline, Temporal, Parietal, Prefrontal, Occipital and Central for Pre-MF Vs Post-MF Rest State

Features	Frontal	Midline	Temporal	Parietal	Prefrontal	Occipital	Central
**Total Alpha Band Power**	Post-MF> Pre-MF (p=2.7540e-07)	Post-MF> Pre-MF (p=1.8566e-12)	Post-MF> Pre-MF (p=2.2479e-10)	Post-MF> Pre-MF (p=7.0730e-13)	Post-MF> Pre-MF (p=1.3501e-06)	Post-MF> Pre-MF (p=4.2558e-10)	Post-MF> Pre-MF (p=1.1731e-12)
**Total Beta Band Power**	Pre-MF> Post-MF (p=2.7286e-16)	Pre-MF> Post-MF (p=2.5889e-12)	Pre-MF> Post-MF (p=7.2110e-21)	Pre-MF> Post-MF (p=5.6585e-14)	Pre-MF> Post-MF (p=1–6111e-14)	Pre-MF> Post-MF (p=3.0201e-31)	Pre-MF> Post-MF (p=3.1421–13)
**Total Power**	(p=0.2780)	Post-MF> Pre-MF (p=5.2237e-05)	Post-MF> Pre-MF (p=0.0132)	Post-MF> Pre-MF (p=6.1665e-07)	Post-MF> Pre-MF (p=0.0147)	(p=1386)	Post-MF> Pre-MF (p=0.0022)
**Total Theta Band Power**	Post-MF> Pre-MF (p= 1.7956e-05)	(p=0.1658)	Post-MF> Pre-MF (p= 0.001)	(p=0.9784)	Post-MF> Pre-MF (p=4.8613e-06)	Post-MF> Pre-MF (p=1.2131e-06)	Post-MF> Pre-MF (p=0.026)
**Relative Theta Band Power**	Post-MF> Pre-MF (p=0.0285)	Pre-MF>Post-MF (p =0.0450)	(p=0.4199)	Pre-MF> Post-MF (p=6.8113e-04)	(p=0.1458)	Post-MF> Pre-MF (p=0.0271)	(p=0.3081)
**Relative Alpha Band Power**	Post-MF> Pre-MF (p=5.3034e-12)	Post-MF> Pre-MF (p=4.4706e-16)	Post-MF> Pre-MF (p=5.2196e-16)	Post-MF> Pre-MF (p=3.8400e-17)	Post-MF> Pre-MF (p=5.1168e-09)	Post-MF> Pre-MF (p=2.5840e-17)	Post-MF> Pre-MF (p=1.5989e-19)
**Relative Beta Band Power**	Pre-MF> Post-MF (p=4.1474e-22)	Pre-MF> Post-MF (p=8.8022e-19)	Pre-MF> Post-MF (p=3.7153e-28)	Pre-MF> Post-MF (p=1.1258e-21)	Pre-MF> Post-MF (p=1.7363e-21)	Pre-MF> Post-MF (p=2.9874e-37)	Pre-MF> Post-MF (p=2.5370e-21)
