# Supplementary material for: Quantitative EEG Changes in Youth With ASD Following Brief Mindfulness Meditation Exercise
Source: IEEE Trans Neural Syst Rehabil Eng. Author manuscript; Available in PMC 2023 Mar 2. (PMC9979338; doi:10.1109/TNSRE.2022.3199151)
Supplement: supp1-3199151 [file NIHMS1834258-supplement-supp1-3199151.pdf]

Quantitative EEG changes in Youth with ASD following brief mindfulness meditation exercise

Busra T. Susam, Nathan T. Riek, Kelly Beck, Safaa Eldeeb, Jane Yun, Caitlin M. Hudac, Philip Gable, Caitlin Conner, Murat Akcakaya, Susan White, Carla Mazefsky

The results of Wilcoxon rank-sum test applied on each spectral feature averaged over each brain regions of frontal , midline, temporal, parietal, prefrontal, occipital and central for (i) TaskRest1 vs Post-MF, (ii) TaskRest1 vs TaskRest2, (iii)Pre-MF vs Post-MF, (iv)Pre-MF-TaskRest2, and (v)Post-MF vs TaskRest2 in Table S1 in the supplementary document.

Table S1: The results of Wilcoxon rank-sum test applied on each spectral feature averaged over each brain regions for (i) TaskRest1 vs Post-MF, (ii) TaskRest1 vs TaskRest2, (iii)Pre-MF vs Post-MF (iv)Pre-MF vs Post-MF, (v)Pre-MF-TaskRest2, and (vi)Post-MF vs TaskRest2

Total Alpha band power	*TaskRest1 vs Pre-MF*	*TaskRest1 vs Post-MF*	*TaskRest1 vs TaskRest2*	*Pre-MF vs Post-MF*	*Pre-MF vs TaskRest2*	*Post-MF vs TaskRest2*
**Frontal**	Pre-MF> TaskRest1, p=1.3441e-07	Post-MF> TaskRest1, p=2.7390e-25	TaskRest2> TaskRest1, p=9.4536e-08	Post-MF> Pre-MF, p=2.7540e-07	p=0.9162	Post-MF> TaskRest2, p=5.4778e-07
**Midline**	Pre-MF> TaskRest1, p=2.5083e-15	Post-MF> TaskRest1, p=1.6187e-50	TaskRest2> TaskRest1, p=3.3193e-08	Post-MF> Pre-MF, p=1.8566e-12	Pre-MF> TaskRest2, p=0.0142	Post-MF> TaskRest2, p=1.0924e-19
**Temporal**	Pre-MF> TaskRest1, p=1.4553e-19	Post-MF> TaskRest1, p=3.3565e-52	TaskRest2> TaskRest1, p=4.0874e-12	Post-MF> Pre-MF, p=2.2479e-10	Pre-MF> TaskRest2, p=0.0199	Post-MF> TaskRest2, p=1.2712e-16

**Parietal**	Pre-MF> TaskRest1, p=1.0774e-17	Post-MF> TaskRest1, p=1.307e-55	TaskRest2> TaskRest1, p=5.2112e-10	Post-MF> Pre-MF, p=7.0730e-13	Pre-MF> TaskRest2, p=0.0094	Post-MF> TaskRest2, p=3.7852e-21
**Prefrontal**	p=0.0633	Post-MF> TaskRest1, p=7.7029e-12	TaskRest2> TaskRest1, p=1.2693e-06	Post-MF> Pre-MF, p=1.3501e-06	TaskRest2>Pre-MF, p=0.0042	Post-MF> TaskRest2, p=0.0147
**Occipital**	Pre-MF> TaskRest1, p=8.6765e-09	Post-MF> TaskRest1, p=1.2468e-31	TaskRest2> TaskRest1, p=0.0018	Post-MF> Pre-MF, p=4.2558e-10	Pre-MF> TaskRest2, p=0.0052	Post-MF> TaskRest2, p=6.4467e-18
**Central**	Pre-MF> TaskRest1, p=2.6498E-26	Post-MF> TaskRest1, p=5.6127e-68	TaskRest2> TaskRest1, p=1.0462e-18	Post-MF> Pre-MF, p=1.1731e-12	p=0.1418	Post-MF> TaskRest2, p=4.9885e-17
Total Beta band power	*TaskRest1 vs Pre-MF*	*TaskRest1 vs Post-MF*	*TaskRest1 vs TaskRest2*	*Pre-MF vs Post-MF*	*Pre-MF vs TaskRest2*	*Post-MF vs TaskRest2*
**Frontal**	Pre-MF> TaskRest1, p=1.6166e-06	TaskRest1> Post-MF, p=1.8860e-05	p=0.1343	Pre-MF> Post-MF, p=2.7286e-16	Pre-MF> TaskRest2, p=0.0036	TaskRest2> Post-MF, p=1.5955e-07
**Midline**	Pre-MF> TaskRest1, p=4.7461e-04	TaskRest1> Post-MF, p=3.6923e-05	TaskRest2> TaskRest1, p=2.8785e-05	Pre-MF> Post-MF, p=2.5889e-12	p=0.4141	TaskRest2> Post-MF, p=3.0660e-14
**Temporal**	Pre-MF> TaskRest1, p=1.2614e-11	TaskRest1> Post-MF, p=4.2985e-04	TaskRest2> TaskRest1, p=3.1287e-12	Pre-MF> Post-MF, p=7.2110e-21	p=0.4045	TaskRest2> Post-MF, p=1.5522e-21
**Parietal**	p=0.4867	TaskRest1> Post-MF, p=3.9028e-13	TaskRest2> TaskRest1, p=2.3076e-04	Pre-MF> Post-MF, p=5.6585e-14	TaskRest2> Pre-MF, p=0.0038	TaskRest2> Post-MF, p=6.0998e-23
**Prefrontal**	p=0.0671	TaskRest1> Post-MF, p=1.4378e-10	p=0.3944	Pre-MF> Post-MF, p=1.6111e-14	p=4224	TaskRest2> Post-MF, p=2.9895e-11

**Occipital**	Pre-MF> TaskRest1, p=1.9325e-25	TaskRest1> Post-MF, p=0.0044	TaskRest2> TaskRest1, p=2.5765e-10	Pre-MF> Post-MF, p=3.0201e-31	Pre-MF> TaskRest2, p=2.0902e-05	TaskRest2> Post-MF, p=2.1620e-14
**Central**	Pre-MF> TaskRest1, p=3.7577e-06	TaskRest1> Post-MF, p=0.0011	TaskRest2> TaskRest1, p=1.2213e-07	Pre-MF> Post-MF, p=3.1421-13	p=0.4082	TaskRest2> Post-MF, p=2.8330e-15
Total Power	*TaskRest1 vs Pre-MF*	*TaskRest1 vs Post-MF*	*TaskRest1 vs TaskRest2*	*Pre-MF vs Post-MF*	*Pre-MF vs TaskRest2*	*Post-MF vs TaskRest2*
**Frontal**	Pre-MF> TaskRest1, p=5.6530e-16	Post-MF> TaskRest1, p=3.8517e-20	TaskRest2> TaskRest1, p=2.1326e-13	p=0.2780	p=0.7697	p=0.1621
**Midline**	Pre-MF> TaskRest1, p=5.0506e-21	Post-MF> TaskRest1, p=8.6676e-42	TaskRest2> TaskRest1, p=1.9334e-14	Post-MF> Pre-MF, p=5.2237e-05	p=0.1814	Post-MF> TaskRest2, p=1.7108e-07
**Temporal**	Pre-MF>TaskRest1, p=1.9505e-30	Post-MF>TaskRest1, p=7.0828e-44	TaskRest2> TaskRest1, p=5.0237e-30	Post-MF> Pre-MF, p=0.0132	p=0.7883	Post-MF> TaskRest2, p=0.0304
**Parietal**	Pre-MF> TaskRest1, p=5.4750e-19	Post-MF> TaskRest1, p=2.9014e-44	TaskRest2> TaskRest1, p=7.7931e-16	Post-MF> Pre-MF, p=6.1665e-07	p=0.4788	Post-MF> TaskRest2, p=1.8298e-08
**Prefrontal**	Pre-MF> TaskRest1, p=6.0228e-05	Post-MF> TaskRest1, p=5.6410e-10	TaskRest2> TaskRest1, p=1.9303e-09	Post-MF> Pre-MF, p=0.0147	TaskRest2> Pre-MF, p=0.0188	p=0.9468
**Occipital**	Pre-MF> TaskRest1, p=9.2844e-28	Post-MF> TaskRest1, p=2.1989e-36	TaskRest2> TaskRest1, p=1.3305e-09	p=0.1386	Pre-MF>TaskRest2, p=1.5605e-06	Post-MF> TaskRest2, p=3.7160e-10
**Central**	Pre-MF> TaskRest1, p=5.8854e-31	Post-MF> TaskRest1, p=1.4238e-48	TaskRest2> TaskRest1, p=8.9950e-27	Post-MF> Pre-MF, p=0.0022	p=0.6042	Post-MF> TaskRest2, p=5.4666e-04

Total Theta band power	*TaskRest1 vs Pre-MF*	*TaskRest1 vs Post-MF*	*TaskRest1 vs TaskRest2*	*Pre-MF vs Post-MF*	*Pre-MF vs TaskRest2*	*Post-MF vs TaskRest2*
**Frontal**	p=0.0551	Post-MF> TaskRest1, p=0.0045	p=0.8512	Post-MF> Pre-MF, p= 1.7956e-05	p=0.1431	Post-MF> TaskRest2, p=0.0066
**Midline**	Pre-MF> TaskRest1, p=0.0286	Post-MF> TaskRest1, p=3.5551e-04	p=0.9799	p=0.1658	p=0.0500	Post-MF> TaskRest2, p=0.0018
**Temporal**	p=0.3626	Post-MF> TaskRest1, p=0.0014	TaskRest1> TaskRest2, p=0.0034	Post-MF> Pre-MF, p= 0.001	Pre-MF> TaskRest2, p=0.0160	Post-MF> TaskRest2, p=6.3922e-07
**Parietal**	Pre-MF> TaskRest1, p=0.0024	Post-MF> TaskRest1, p=0.0030	p=0.4280	p=0.9784	p=0.0870	p=0.0581
**Prefrontal**	p=0.9749	Post-MF> TaskRest1, p=7.4305e-07	p=0.2995	Post-MF> Pre-MF, p= 4.8613e-06	p=0.3587	Post-MF> TaskRest2, p=6.024e-04
**Occipital**	p=1	p=0.6596	p=0.0635	Post-MF> Pre-MF, p= 1.2131e-06	TaskRest2> Pre-MF, p= 4.7261e-05	p=0.2128
**Central**	Pre-MF> TaskRest1, p=0.0118	Post-MF> TaskRest1, p=6.6168e-06	p=0.7469	Post-MF> Pre-MF, p= 0.026	p=0.0584	Post-MF> TaskRest2, p=4.0523e-04
Relative Theta band power	*TaskRest1 vs Pre-MF*	*TaskRest1 vs Post-MF*	*TaskRest1 vs TaskRest2*	*Pre-MF vs Post-MF*	*Pre-MF vs TaskRest2*	*Post-MF vs TaskRest2*
**Frontal**	TaskRest1> Pre-MF, p=2.8890e-11	TaskRest1> Post-MF, p=2.8114e-06	TaskRest1> TaskRest2, p=6.3951e-05	Post-MF> Pre-MF, p=0.0285	TaskRest2> Pre-MF, p=0.0118	p=0.6802

**Midline**	TaskRest1> Pre-MF, p=6.3812e-09	TaskRest1> Post-MF, p=5.1378e-14	TaskRest1> TaskRest2, p=1.1975e-05	Pre-MF> Post-MF, p=0.0450	p=0.2145	TaskRest2> Post-MF, p=0.0028
**Temporal**	TaskRest1> Pre-MF, p=4.3076e-15	TaskRest1> Post-MF, p=1.2238e-12	TaskRest1> TaskRest2, p=6.5203e-14	p=0.4199	p=0.9255	p=0.028
**Parietal**	TaskRest1> Pre-MF, p=4.9433e-06	TaskRest1> Post-MF, p=2.7155e-15	TaskRest1> TaskRest2, p=2.4082e-05	Pre-MF> Post-MF, p=6.8113e-04	p=0.7728	TaskRest2> Post-MF, p=2.7047e-04
**Prefrontal**	TaskRest1> Pre-MF, p=0.0148	p=0.5124	TaskRest1> TaskRest2, p=0.0097	p=0.1458	p=0.8302	p=0.0530
**Occipital**	TaskRest1> Pre-MF, p=7.7433e-21	TaskRest1> Post-MF, p=6.1375e-14	TaskRest1> TaskRest2, p=3.1449e-05	Post-MF> Pre-MF, p=0.0271	TaskRest2> Pre-MF, p=1.2884e-07	TaskRest2> Post-MF, p=5.2278e-04
**Central**	TaskRest1> Pre-MF, p=6.9395e-15	TaskRest1> Post-MF, p=9.1549e-19	TaskRest1> TaskRest2, p=1.4275e-12	p=0.3081	p=0.6803	p=0.1913
Relative Alpha band power	*TaskRest1 vs Pre-MF*	*TaskRest1 vs Post-MF*	*TaskRest1 vs TaskRest2*	*Pre-MF vs Post-MF*	*Pre-MF vs TaskRest2*	*Post-MF vs TaskRest2*
**Frontal**	Pre-MF> TaskRest1, p=0.0034	Post-MF> TaskRest1, p=2.1617e-23	TaskRest2> TaskRest1, p=4.1964e-04	Post-MF> Pre-MF, p=5.3034e-12	p=0.5767	Post-MF> TaskRest2, p=1.4121e-10
**Midline**	Pre-MF> TaskRest1, p=1.6863e-09	Post-MF> TaskRest1, p=1.2502e-45	TaskRest2> TaskRest1, p=8.6554e-04	Post-MF> Pre-MF, p=4.4706e-16	Pre-MF> TaskRest2, p=0.0041	Post-MF> TaskRest2, p=2.4106e-26
**Temporal**	Pre-MF> TaskRest1, p=2.1247e-09	Post-MF> TaskRest1, p=5.2484e-44	TaskRest2> TaskRest1, p=0.0041	Post-MF> Pre-MF, p=5.2196e-16	Pre-MF> TaskRest2, p=9.9103e-04	Post-MF> TaskRest2, p=8.6563e-28

**Parietal**	Pre-MF> TaskRest1, p=9.9512e-13	Post-MF> TaskRest1, p=1.2498e-53	TaskRest2> TaskRest1, p=1.0579e-04	Post-MF> Pre-MF, p=3.8400e-17	Pre-MF> TaskRest2, p=5.6218e-04	Post-MF> TaskRest2, p=2.1658e-30
**Prefrontal**	p=0.8924	Post-MF> TaskRest1, p=3.442e-10	TaskRest2> TaskRest1, p=0.0016	Post-MF> Pre-MF, p=5.1168e-09	TaskRest2> Pre-MF, p=0.0043	Post-MF> TaskRest2, p=4.3073e-04
**Occipital**	p=0.5068	Post-MF> TaskRest1, p=1.1493e-20	p=0.7865	Post-MF> Pre-MF, p=2.5840e-17	p=0.6980	Post-MF> TaskRest2, p=6.0312e-19
**Central**	Pre-MF> TaskRest1, p=1.3520e-15	Post-MF> TaskRest1, p=6.7715e-64	TaskRest2> TaskRest1, p=1.14588e-09	Post-MF> Pre-MF, p=1.5989e-19	p=0.0737	Post-MF> TaskRest2, p=1.0021E-26
Relative Beta band power	*TaskRest1 vs Pre-MF*	*TaskRest1 vs Post-MF*	*TaskRest1 vs TaskRest2*	*Pre-MF vs Post-MF*	*Pre-MF vs TaskRest2*	*Post-MF vs TaskRest2*
**Frontal**	Pre-MF> TaskRest1, p=4.5053e-04	TaskRest1> Post-MF, p=2.6003e-13	p=0.7195	Pre-MF> Post-MF, p=4.1474e-22	Pre-MF> TaskRest2, p=0.0056	TaskRest2> Post-MF, p=7.6099e-12
**Midline**	p=0.7293	TaskRest1> Post-MF, p=8.1676e-20	TaskRest2> TaskRest1, p=0.0043	Pre-MF> Post-MF, p=8.8022e-19	TaskRest2> Pre-MF, p=0.0169	TaskRest2> Post-MF, p=2.3669e-26
**Temporal**	Pre-MF> TaskRest1, p=5.3466e-05	TaskRest1> Post-MF, p=1.5455e-16	TaskRest2> TaskRest1, p=2.4597e-07	Pre-MF> Post-MF, p=3.7153e-28	p=0.1205	TaskRest2> Post-MF, p=4.8496e-31
**Parietal**	TaskRest1> Pre-MF, p=0.0066	TaskRest1> Post-MF, p=7.9517e-36	TaskRest2> TaskRest1, p=0.0448	Pre-MF> Post-MF, p=1.1258e-21	TaskRest2> Pre-MF, p=6.5550e-05	TaskRest2> Post-MF, p=7.6710e-37
**Prefrontal**	p=0.2668	TaskRest1> Post-MF, p=5.4660e-20	p=0.6950	Pre-MF> Post-MF, p=1.7363e-21	p=0.1793	TaskRest2> Post-MF, p=2.4422e-15

**Occipital**	Pre-MF> TaskRest1, p=1.1186-13	TaskRest1> Post-MF, p=3.2884e-13	TaskRest2> TaskRest1, p=7.9398e-07	Pre-MF> Post-MF, p=2.9874e-37	Pre-MF> TaskRest2, p=0.0024	TaskRest2> Post-MF, p=3.8477e-24
**Central**	Pre-MF> TaskRest1, p=0.0312	TaskRest1> Post-MF, p=2.5605e-17	TaskRest2> TaskRest1, p=0.0015	Pre-MF> Post-MF, p=2.5370e-21	p=0.2721	TaskRest2> Post-MF, p=2.9318e-24

The performance measurements of Pre-MF and Post-MF classification by the RBF SVM classifier are presented for each participant in Table S2 in the supplementary document

Table S2. The classification results of before (Pre-MF) and after mindfulness exercise (Post-MF).

ID	Accuracy	Sensitivity	Specificity	F1 Score
1	72.57	41.33	89.15	0.50
2	72.05	70.00	74.12	0.71
3	77.33	74.59	80.11	0.77
4	81.50	74.08	88.91	0.80
5	79.72	79.23	80.20	0.79
6	89.43	91.27	87.57	0.90
7	65.89	64.17	67.60	0.65
8	68.87	68.66	69.08	0.68
9	79.64	82.58	76.75	0.80
10	79.23	80.71	77.77	0.79
11	90.40	79.65	96.11	0.85
12	86.36	64.53	96.84	0.75
13	75.69	52.27	90.04	0.62
14	81.51	83.87	80.26	0.76
15	96.36	96.84	96.11	0.95
16	76.09	78.32	73.86	0.77
17	71.93	72.21	71.65	0.72
18	73.28	70.95	75.67	0.73
19	85.89	91.98	79.70	0.87
20	78.65	69.83	87.62	0.76
21	82.65	86.68	78.57	0.83
22	85.48	76.67	94.45	0.84
23	81.30	78.34	84.32	0.81
24	88.05	91.77	84.28	0.89
25	72.64	67.43	77.70	0.71
26	88.20	90.23	86.15	0.89
27	79.85	82.35	77.16	0.81
28	95.84	97.54	93.97	0.96
29	77.29	77.74	76.85	0.78
30	76.51	77.75	75.18	0.77
31	95.05	97.74	92.19	0.95
32	82.87	86.09	79.45	0.84
33	80.52	83.46	77.36	0.82
34	79.45	82.48	76.24	0.80
35	78.45	75.05	82.02	0.78
Mean± STD	80.76± 7.28	78.24± 11.88	82.14± 7.88	0.79± 0.09

The distribution of the selected features at each channel location for each individual for Pre-MF and Post-MF classification problem is shown in the supplementary Table S3.

Table S3 : The most informative feaures of the classification of Pre-MF vs Post-MF.

Participant	'Relative Power in Beta band'	Relative Power in Alpha band'	Relative Power in Theta band'	'Total Power in Beta band'	Total Power in Alpha band'	'Total Power in Theta band'	'Total power'
1	x	x	Cz	F8	x	O2	F7
2	O2	x	x	O1	x	O2	x
3	T3	O2	F4,T4	x	x	x	x
4	T7	Fp1	x	T5,O2	O2,Pz	O2	x
5	x	F4	x	F7	x	x	F4,Cz
6	x	x	x	P3,O2,T6	x	x	x
7	x	x	x	C3	O1	P4	Fp1
8	O1,O2,T6	x	x	F3	x	Fp1	Cz,O1
9	Fp2,T6	x	O2	x	x	x	F4
10	x	Pz	x	x	x	P4	T3,Pz
11	F8	T5	x	x	F7	Fp2	x
12	C3	x	x	C3,O1	x	x	P3
13	x	Pz	Fp2	Pz	x	F4	x
14	x	T6	x	x	T6	x	Fp1
15	F7	x	x	Fz,T5,F8,T6	P3	x	x
16	C3,Cz	C3,F3,T3	x	O2	Fp1	x	x
17	P4,Pz	F3	x	x	F3	x	x
18	x	P3,O2	x	x	C3	x	x
19	T3	x	Fp1	C3,Fz,T5	P3	F4	Pz
20	O1	P3,Fz,T5	O1	x	Fz,Fp2,O1	T5	Pz
21	O1,O2,T6	T6	x	T6	x	Fp1	F3
22	O2	x	x	x	x	x	O1,O2
23	C3	x	x	C3	F3	C3	x
24	x	x	F3	F3,F5	x	x	T5, O2
25	x	T6	x	x	x	O2	x
26	x	x	T5	T3,O2	x	x	P4,Pz
27	x	O1	x	F8	x	C4	x
28	x	C4,T3,O1,T4	T3	x	x	x	x
29	x	x	T3,O2	T4	C4	x	x
30	F7	x	x	T3,O1	x	x	x
31	C3,O2,F8	C3	x	x	x	x	x
32	F8	C3	x	x	x	x	x
33	T3	F8	x	P3	x	x	x
34	T6	x	C4	x	F8	x	F8
35	T6	x	x	x	O1	x	x
